# Epidemiology and healthcare utilization of First Nations peoples living with spinal cord injury in Alberta: an observational study to explore health inequities

**DOI:** 10.1038/s41394-023-00603-4

**Published:** 2023-09-08

**Authors:** Brett F. Wegenast, Tara A. Whitten, Jeffrey A. Bakal, Lea Bill, Adalberto Loyola-Sanchez

**Affiliations:** 1 University of Alberta, Edmonton, AB Canada; 2 Alberta Health Services, Edmonton, AB Canada; 3 Alberta First Nations Information Governance Center, Edmonton, AB Canada

**Keywords:** Rehabilitation, Epidemiology

## Abstract

**Study design:**

Retrospective observational cohort study.

**Objectives:**

Estimate spinal cord injury (SCI) prevalence in First Nations and non-First Nations populations and compare healthcare utilization as an indirect marker of health inequities.

**Setting:**

Alberta, Canada.

**Methods:**

We created a prevalent adult SCI cohort by identifying cases between April 1, 2002 and December 31, 2017 who were followed for common SCI complications and location of healthcare access from January 1, 2018 to December 31, 2019 using administrative data sources housed within Alberta Health Services (AHS). First Nations and non-First Nations SCI cohorts were divided into SCI etiology: traumatic SCI (TSCI) and non-traumatic SCI (NTSCI). Statistical analyses compared prevalence, demographics, healthcare utilization, and SCI complication rates. A secondary analysis was performed using case matching for demographics, injury type, injury level, and comorbidities.

**Results:**

TSCI prevalence: 248 and 117 per 100,000 in First Nations and non-First Nations cohorts, respectively. NTSCI prevalence: 74 and 50 per 100,000 in First Nations and non-First Nations cohorts, respectively. Visit rates were higher in the TSCI First Nations cohort for visits to General Practitioner (GP), Emergency Department (ED), inpatient visits, and inpatient days with higher complication rates due to pulmonary, genitourinary, skin, and ‘other’ causes after case matching. Visits rates were higher in the NTSCI First Nations cohort for GP and specialists without differences in complication types after case matching.

**Conclusions:**

Significant differences exist between First Nations and non-First Nations cohorts living with SCI in Alberta, suggesting healthcare inequities against First Nations Peoples in this province.

## Introduction

Spinal cord injury (SCI) is any damage to the spinal cord that causes temporary or permanent loss of function, which often results in severe and permanent disability. In Alberta, the annual incidence of traumatic SCI is approximately 52 per million, with a survival rate of 85% [1]. Considering Alberta’s current population of 4.3 million people [2], there is an estimate of 190 traumatic SCI survivors per year in this province.

The most common causes of traumatic SCI in Alberta are motor vehicle accidents (56.4%) and falls (19.1%) [1]. Major risk factors for SCI include being a young adult male, rural location, and using alcohol [1, 3]. After surviving the first year after SCI, patients have a life expectancy of approximately 90% of the average lifespan of the non-injured population [4], resulting in a significant number of people living with SCI as a chronic condition.

Living with SCI is associated with multiple physical and psychological complications, many of which are common and potentially severe. After SCI, 55% of patients will be re-hospitalized within the first year and 37% will be re-hospitalized per year afterwards [5]. Common health problems after SCI include complications of the urinary system, gastrointestinal system, bone metabolism, muscle spasticity, joint contractures, pressure ulcers, pain, and mental health problems including depression, addiction, and suicide. These chronic complications contribute to reduced quality of life and substantial costs to the healthcare system estimated to be between $500,000 and 2 million dollars over the lifetime of a single SCI patient [6].

Indigenous populations are known to have disparities in access to healthcare services throughout the world and Canada is no exception. Barriers to healthcare access include rural location, language, and socioeconomic status, which in Canada are frequently associated with social, historical, and political forces that produce disadvantages to Indigenous Peoples [7, 8]. This contributes to Indigenous populations living with higher rates of chronic illnesses such as hypertension, obesity, diabetes, cardiovascular disease, and autoimmune rheumatologic conditions [9, 10]. In Alberta, there are over 258,000 Indigenous Peoples with more than half of them living in rural locations or small population centers [11]. Among the Indigenous populations in Alberta nearly 137,000 identify as First Nations Peoples and approximately 112,000 are Registered or Treaty Indian [12].

Life expectancy of Indigenous Peoples in Alberta is 10 years shorter than the non-Indigenous population, and this is related to higher prevalence of infections and chronic diseases [13, 14]. In addition, Indigenous Peoples commonly experience higher acute care needs than their non-Indigenous counterparts as evidenced by higher utilization rates of emergency health services in this province [15]. Considering the high rate of complications associated with living with a SCI and the known inequities that Indigenous Peoples face in Alberta, it is justified to suspect that Indigenous Peoples with SCI in Alberta are facing important health inequities in comparison to their non-Indigenous counterparts. Consequently, the objectives of this study were: 1) to compare the prevalence of traumatic SCI (TSCI) and non-traumatic SCI (NTSCI) between First Nations and non-First Nations populations in Alberta, Canada and 2) to explore health inequities that negatively impact the lives of First Nations Peoples through comparing healthcare utilization rates between these groups.

## Methods

### Study setting and data sources

We conducted a retrospective observational cohort study in Alberta, a Canadian province with a single publicly funded universal healthcare system. Six administrative data sources housed within Alberta Health Services, the corporation that manages most of the healthcare services in the province, were used for this study along with the utilization of diagnostic codes from the International Statistical Classification of Diseases and Related Health Problems, 10th revision, with Canadian enhancements (ICD-10-CA). The six administrative databases consulted were: 1) The Discharge Abstract Database (DAD), which tracks all acute care inpatient discharges from Alberta hospitals, 2) The National Ambulatory Care Reporting System (NACRS) that tracks all Emergency Department (ED) visits, 3) The Practitioner Claims database, which tracks all health service claims submitted for payment by healthcare providers using the International Statistical Classification of Diseases and Related Health Problems, 9th revision (ICD-9), 4) The National Rehabilitation System (NRS) database that tracks inpatient rehabilitation stays, 5) The Provincial Registry that identifies date of death and Alberta Health Care Insurance Plan (AHCIP) coverage for Alberta Residents, and 6) The Vital Statistics database, which also tracks dates of death of Alberta residents. This study received ethics approval and a waiver of informed consent from the University of Alberta Health Research Ethics Board (Pro00097538).

### Study cohort

We created a prevalent SCI cohort by looking for cases between April 1, 2002 and December 31, 2017 who were over 18 years of age, alive, and residents of Alberta on January 1, 2018 using a validated administrative case definition with some modifications [16]. This population was then followed for outcomes until December 31, 2019. Two main cohorts were created, one for traumatic etiologies (i.e., TSCI cohort) and one for non-traumatic etiologies (i.e., NTSCI cohort) as identified using ICD-10-CA diagnosis codes (Supplementary Table 1). In cases where individuals met both TSCI and NTSCI case definitions they were classified according to which definition they met first. Individuals were excluded from the study if they were younger than 18 on January 1, 2018, if they died before this date, were not Alberta residents (based on the Provincial Registry), or if they did not have provincial healthcare coverage for any time during the follow-up period. Prevalence was calculated as of January 1, 2018. Mortality rates were estimated based on deaths during the two-year follow-up period from January 1, 2018 to December 31, 2019. Those who died during the follow-up period were included for prevalence and mortality rate estimates, but excluded from the follow-up outcomes (visit and complication rates).

Individuals were flagged as First Nations or non-First Nations by the Alberta Ministry of Health based on the First Nations Status Registry, which includes all AHCIP registrants associated with a First Nations group number since 1983 and individuals who register under a main AHCIP account for which the main registrant had a First Nations group number. First Nations status is given to all individuals who are on the First Nations Status Registry at the mid-year registry file (June 30 each year).

### Covariates

The Charlson comorbidity index was calculated using Deyo weights [17, 18] by reviewing 2 years prior to the index date in DAD, NACRS, and Practitioner Claims where comorbidities identified in Claims required at least two visits with the appropriate diagnostic code. All individuals in the cohort were assigned 2 points in the Charlson index for the comorbidity of SCI. Location of residence was based on the Alberta Health Services classification of sub-local geographic areas as Metropolitan, Urban, or Rural.

### Outcome measures

We quantified healthcare utilization and the occurrence of common SCI complications during the 2-year follow-up period from January 1, 2018 to December 31, 2019. Healthcare utilization measures included the number of visits to a general practitioner (GP), specialist, ED, inpatient visits, and inpatient days. GP and specialist visits were based on unique physician-days from the Practitioner Claims database in a community setting (physician office, clinic, or long-term care delivery site). Specialist visits were limited to those with SCI-related practitioner specialties and included: Physical Medicine and Rehabilitation, Orthopedic Surgery, Urology, Cardiology, Respiratory Medicine, Nephrology, General Surgery, Infectious Diseases, Neurology, Neurosurgery, Plastic Surgery, Gastroenterology, and Podiatry. The occurrence of SCI-related complications were quantified for community, emergency, and inpatient settings. Complications were grouped as pulmonary, gastrointestinal (GI), genitourinary (GU), skin, cardiovascular/autonomic, and other (Supplementary Table 2).

### Analysis

We compared baseline characteristics between First Nations and non-First Nations groups. TSCI and NTSCI were analyzed separately, using a *t*-test for continuous variables and a chi-square test for categorical variables to compare First Nations versus non-First Nations populations. Prevalence ratios and mortality rate ratios were estimated using a log-binomial model. The conditional mean is the median number of visits (Table 2) or complications (Table 3) of those who had at least one visit/complication. Rate ratios and associated *p* values comparing First Nations and non-First Nations healthcare utilization and the occurrence of complications were estimated using a negative binomial model. Adjusted rate ratios were calculated by including the following predictors in the model: injury type (TSCI/NTSCI), residence (metropolitan/urban/rural), sex, age (under 40/40–65/65+years), level of injury (paraplegic/quadriplegic/unknown) and Charlson index (Mild: ≤2/Moderate: 2–4/Severe: ≥5).

A secondary analysis using matched First Nations and non-First Nations cohorts was performed. Non-First Nations controls were matched to the First Nations cohort with a ratio of 2:1 using exact match on sex, residence location (metropolitan/urban/rural), injury type (TSCI/NTSCI), injury level (tetraplegia/paraplegia/unknown), age +/−5 years and Charlson index +/−2. A greedy matching algorithm was used so those with the closest matches were selected. Chi-square testing was used to determine significant differences between First Nations and non-First Nations cohorts when data values were at least 5 and a Fisher’s exact test was used when values were less than 5. All statistical analyses were performed using SAS 9.4 (SAS Institute, Cary, NC).

## Results

### Prevalence of SCI among First Nations and non-First Nations peoples

We identified that Alberta’s population over 18 years of age at the beginning of the two-year follow-up period (i.e., January 1, 2018) was approximately 111,190 for First Nations (i.e., First Nations cohort) and 3,284,141 for non-First Nations (i.e., non-First Nations cohort) people. The prevalence of First Nations Peoples living with TSCI in Alberta was twice as large compared to non-First Nations with a prevalence of 248 and 117 per 100,000, respectively (Prevalence Ratio [PR] 2.1, 95% CI 1.9–2.4). Similarly, the prevalence of NTSCI was higher in the First Nations group at 74 per 100,000 compared to non-First Nations at 50 per 100,000 (PR 1.5 95%, CI 1.2–1.8). During the 2-year follow-up period there was no significant difference in mortality rates between the groups.

### Demographics

The average age of individuals living with SCI was significantly younger in the First Nations cohort, being approximately 9 years younger for those living with TSCI and 7 years younger for those living with NTSCI compared to their non-First Nations counterparts (Table 1). The rates of SCI were higher in males for both TSCI and NTSCI etiology, but there was no difference between the First Nations and non-First Nations cohorts based on sex. The majority of First Nations Peoples living with SCI lived in rural locations compared to non-First Nations individuals, who primarily lived in metropolitan areas. First Nations individuals living with NTSCI were found to have a higher number of comorbidities when compared to non-First Nations individuals, but there was no difference between the cohorts living with TSCI. The most common etiology for NTSCI in our cohort was degenerative spine disease. Degenerative spine disease was the most common reason for NTSCI in our cohort in both the First Nations (35%) and non-First Nations (45%) groups. The prevalence of congenital and vascular/inflammatory spine diseases as the etiology for NTSCI was identical in both First Nations and non-First Nations groups at 11% and 10%, respectively. The only difference observed in the NTSCI etiology was infectious disease, which was higher in the First Nations group (17%) than in the non-First Nations group (8%).

**Table 1:** Demographics for identified SCI participants living in Alberta January 1, 2018.

		Traumatic SCI			Non-traumatic SCI		
		Non-First Nations (*N* = 3721)	First Nations (*N* = 262)	*p*-value	Non-First Nations (*N* = 1524)	First Nations (*N* = 72)	*p*-value
Age (SD)		51.6 (17.2)	42.5 (14.0)	<0.0001	57.0 (17.0)	49.7 (14.6)	0.0004
Sex	Male	2295 (61.7%)	171 (65.3%)	0.25	884 (58.0%)	44 (61.1%)	0.60
	Female	1426 (38.3%)	91 (34.7%)		640 (42.0%)	28 (38.9%)	
Residence location	Metropolitan	2007 (53.9%)	79 (30.2%)	<0.0001	867 (56.9%)	24 (33.3%)	<0.0001
	Urban	781 (21.0%)	38 (14.5%)		312 (20.5%)	10 (13.9%)	
	Rural	933 (25.1%)	145 (55.3%)		345 (22.6%)	38 (52.8%)	
Level of injury	Paraplegic	1529 (41.1%)	114 (43.5%)	0.54	1095 (71.9%)	52 (72.2%)	0.89
	Tetraplegic	2129 (57.2%)	142 (54.2%)		370 (24.3%)	18 (25.0%)	
	Unknown	63 (1.7%)	6 (2.3%)		59 (3.9%)	2 (2.8%)	
Charlson score^a^ (SD)		2.6 (1.3)	2.6 (1.2)	0.74	3.1 (1.8)	3.6 (2.0)	0.01

^a^Minimum Charlson score is 2 as everyone is assigned the paraplegia/tetraplegia comorbidity. *P* values come from chi-square tests for binary or categorical variables and from *t*-test for continuous variables.

### Number of healthcare visits

Table 2 shows the number of visits to healthcare providers after case matching for demographics and comorbidities (data prior to case matching is available in Supplementary Table 3). For those with TSCI, there was no difference in number of visits to specialists between the First Nations and non-First Nations cohorts, but all other visit types were significantly higher in the First Nations cohort with approximately twice as many visits to a GP and ED. Furthermore, the First Nations cohort with TSCI has approximately three times as many inpatient visits and nearly six times the number of inpatient hospitalization days compared to the non-First Nations cohort with TSCI. Results after case matching comparing the First Nations and non-First Nations cohorts with NTSCI were only significant for the number of visits to GP and specialists with rate ratios estimates of 1.4 (95% CI 1.1–1.9) and 1.6 (95% CI 1.0–2.6), respectively. No significant differences were found for ED, inpatient visits, or inpatient days between the First Nations and non-First Nations cohorts with NTSCI.

**Table 2:** Number of visits to healthcare providers after controlling for demographics and comorbidities.

TSCI	Non-First Nations (*N* = 509)		First Nations (*N* = 258)			
	*N* (%) with at least one visit	Conditional median (IQR)	*N* (%) with at least one visit	Conditional median (IQR)	Rate ratio estimate^a^	*p*-value^a^
GP visits	446 (88%)	8 (3,16)	231 (90%)	15 (8,31)	1.9 (1.6–2.3)	<.0001
Specialist visits	250 (49%)	3 (2,7)	138 (53%)	3 (1,8)	1.2 (0.9–1.6)	0.20
Emergency visits	278 (55%)	2 (1,4)	202 (78%)	4 (2,8)	2.4 (1.9–3.1)	<0.0001
Inpatient visits	87 (17%)	1 (1,2)	100 (39%)	2 (1,3)	3.2 (2.2–4.4)	<0.0001
Inpatient days	87 (17%)	6 (3,15)	100 (39%)	9 (5,32)	5.8 (3.2–10.5)	<0.0001

NTSCI	Non-First Nations (*N* = 131)		First Nations (*N* = 67)			
GP visits	123 (94%)	13 (7,24)	64 (96%)	20 (11,37)	1.4 (1.1–1.9)	0.01
Specialist visits	98 (75%)	5 (2,10)	48 (72%)	4 (2,7)	1.6 (1.0–2.6)	0.04
Emergency visits	79 (60%)	3 (1,7)	52 (78%)	5 (2,13)	1.6 (0.9–2.6)	0.08
Inpatient visits	44 (34%)	2 (1,3)	35 (52%)	2 (1,4)	1.6 (0.9–2.8)	0.08
Inpatient days	44 (34%)	13 (6,48)	35 (52%)	16 (5,54)	1.5 (0.6–3.8)	0.39

^a^Rate ratios and associated *p* values comparing visit rates between First Nations and non-First Nations populations were estimated through negative binomial modelling.

### Complications

A comparison of SCI-related complication rates after case matching is shown in Table 3. The “other” category includes ICD codes related to cramp, spasm, or abnormal muscle movement. A full list of diagnostic codes for SCI-related complications can be found in Supplementary Table 2 and data prior to case matching in Supplementary Table 4. In those with TSCI, the First Nations cohort was found to have significantly more complications than the non-First Nations cohort due to pulmonary, genitourinary, skin, and other diagnoses, with genitourinary diagnoses affecting the highest number of individuals. In the NTSCI population there was no significant difference in the rate of complications in any category between First Nations and non-First Nations cohorts. Similarly, the most common complications for this group were genitourinary.

**Table 3:** Rates of SCI complications after case matching for demographics and comorbidities.

TSCI	Non-First Nations (*N* = 509)		First Nations (*N* = 258)			
Complication	*N* (%) with a complication	Conditional median (IQR)	*N* (%) with complication	Conditional median (IQR)	Rate ratio estimate^a^	*p*-value^a^
Pulmonary	34 (7%)	1 (1,2)	37 (14%)	1 (1,3)	3.3 (1.8–6.1)	0.0001
Gastrointestinal	40 (8%)	1 (1,2)	28 (11%)	1 (1,2)	1.1 (0.6–2.1)	0.72
Genitourinary	133 (26%)	2 (1,5)	100 (39%)	3 (2,8)	1.9 (1.3–2.8)	0.002
Skin	67 (13%)	2 (1,4)	61 (24%)	3 (1,6)	3.0 (1.8–5.1)	<0.0001
Cardiovascular/autonomic	10 (2%)	2 (1,5)	9 (3%)	1 (1,2)	1.1 (0.3–3.9)	0.88
Other	8 (2%)	1 (1,1)	8 (3%)	1 (1,2)	3.2 (1.1–9.6)	0.04

NTSCI	Non-First Nations (*N* = 131)		First Nations (*N* = 67)			
Pulmonary	15 (11%)	2 (1,5)	18 (27%)	1 (1,3)	1.3 (0.5–3.5)	0.63
Gastrointestinal	15 (11%)	1 (1,3)	11 (16%)	2 (1,3)	1.6 (0.6–4.2)	0.39
Genitourinary	60 (46%)	3 (1,7)	35 (52%)	3 (2,8)	1.5 (0.8–2.7)	0.18
Skin	28 (21%)	4 (1,9)	27 (40%)	2 (1,3)	1.7 (0.7–4.1)	0.25
Cardiovascular/autonomic	11 (8%)	1 (1,2)	6 (9%)	1 (1,4)	1.4 (0.4–4.8)	0.58
Other	3 (2%)	1 (1,1)	1 (1%)	1 (1,1)	0.7 (0.1–6.3)	0.71

^a^Rate ratios and associated *p* values comparing complication rates between First Nations and non-First Nations populations were estimated through negative binomial modelling.

**Fig. 1:**
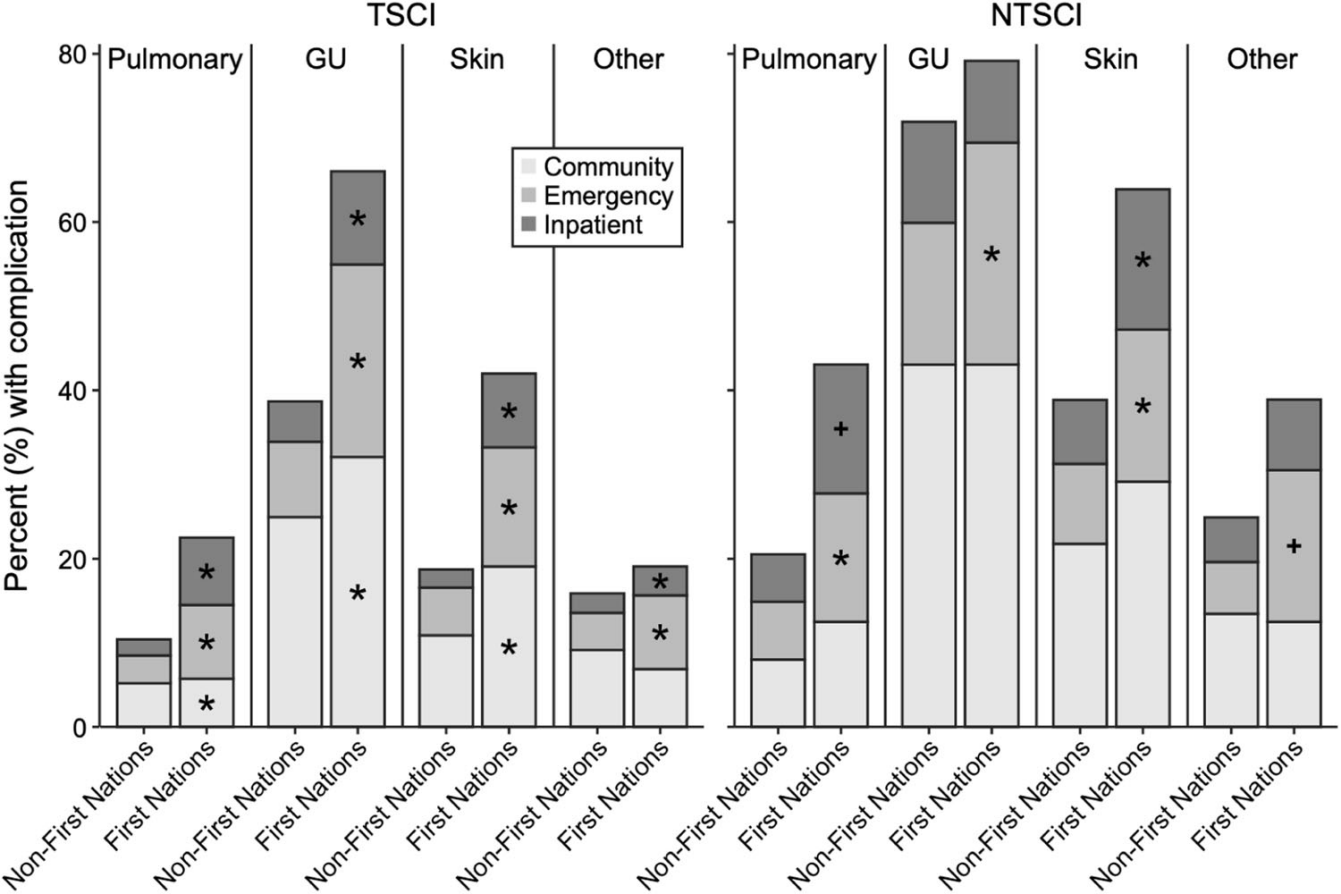
Rate of common SCI complications and type of healthcare access. Rate of common SCI complications and type of healthcare access divided into community (GP and Specialists), ED, and inpatient hospitalizations. GU=genitourinary. * Significantly different from the non-First Nations cohort based on Chi-square test. + Significantly different from the non-First Nations cohort based on a Fisher’s exact test.

A comparison of the most common complications identified in the cohorts within the settings of community (i.e., GP and specialist), ED, and inpatient hospitalizations after case matching is shown in Fig. 1. Categories of complications were divided into pulmonary, genitourinary, skin, while all other diagnoses were aggregated as “other”. Detailed information of all complications and access to healthcare providers can be found in Supplementary Tables 5 and 6, which show data before and after case matching. Within those with TSCI, the First Nations cohort had more visits to community providers, ED, and inpatient hospitalizations for all complications. The differences were less pronounced in the NTSCI population when comparing First Nations to non-First Nations cohorts, but showed a similar trend. First Nations individuals with NTSCI more often required ED care than non-First Nations individuals for all complications assessed. In those with NTSCI, diagnoses relating to pulmonary and skin complications had higher rates of inpatient hospitalization in the First Nations cohort compared to the non-First Nations cohort, but no further statistical differences were found between the cohorts for complications requiring care in the community.

## Discussion

This study estimated the prevalence of SCI in First Nations and non-First Nations populations in Alberta and used access and utilization of healthcare services to serve as an indirect marker of health inequities. At the beginning of the follow-up period, we found a higher prevalence of TSCI and NTSCI among First Nations Peoples in Alberta. Rates of healthcare utilization were higher in the First Nations cohort and were most pronounced in the TSCI group due to higher rates of SCI-related medical complications that require access to community and hospital-based care. This suggests a case of health inequities against First Nations Peoples in this province.

Dryden et al. (2003) previously estimated the incidence of TSCI in Alberta to be approximately 52 per million people per year [1]. This study is the first to investigate the prevalence of SCI in First Nations Peoples of Alberta and to assess potential health inequities faced by these individuals. Our findings confirmed anecdotal evidence experienced by contributors to this study, who have identified higher rates of First Nations Peoples requiring inpatient SCI rehabilitation than expected based on this population’s size. In addition, the significantly higher rates of TSCI and NTSCI in the First Nations cohort follows a similar trend identified in prior literature that shows higher rates of infection and chronic diseases observed in Indigenous Peoples in this province [13, 14]. The only difference observed in the NTSCI etiology among these groups in this study was infectious disease, which was higher in the First Nations group (17%) than in the non-First Nations group (8%). These findings do not clearly show a cause of structural determinants of health regarding the prevalence NTSCI against First Nations Peoples, but suggest a tendency for First Nations Peoples to present with severe spine infection, which could be associated with poorly controlled chronic diseases such as diabetes.

Previously identified major risk factors for TSCI in Alberta include being a young adult male, rural location, and alcohol abuse [1]. Greater than half of the First Nations population in Alberta lives in rural areas, which may contribute to the higher SCI prevalence observed in the First Nations group compared to the non-First Nations group for both TSCI and NTSCI in these locations. Residence location in the First Nations cohort defined in this study (i.e., 55.3% of TSCI and 52.8% of NTSCI First Nations cohorts lived in rural areas) is comparable to that of the 2016 Canadian National census data, in which 52.6% of Indigenous Peoples lived in a rural or small population centers [19], which validates our strategy to define the First Nations cohorts in this study.

According to Statistics Canada’s self-reported health indicator profiles in 2016, rates of binge alcohol consumption in First Nations Peoples were higher than their non-Indigenous counterparts (i.e., 25.1% vs 19.6%) [20]. Moreover, the 2016 Statistics Canada Census found the average age of First Nations Peoples in Alberta to be 28.1 years compared to 37.8 years for those without Indigenous identity [12], which is similar to the age gap we observed between the First Nations and non-First Nations cohort (i.e., 9 years younger in the TSCI and 7 years younger in the NTSCI cohorts). Therefore, the high prevalence of SCI observed in the First Nations population of Alberta could be associated with the known differences between Indigenous and non-Indigenous Peoples in Canada regarding age, residence location, and binge alcohol consumption behaviours. Considering it is well known that addictions in Indigenous communities are the direct result of the intergenerational trauma suffered by Indigenous Peoples in Canada, it is appropriate to suggest that the observed higher rates of TSCI in the Indigenous cohort in this study demonstrates a health inequity conditioned by remote structural determinants of health. This socio-cultural determinant of health is explained by in depth pathological social dynamics that are reproduced by current social structures and result in higher rates of poverty and lack of education or opportunities to climb the social ladder for Indigenous Peoples. This ultimately results in increased risk of addictions that may lead Indigenous persons to incur in risky behaviours associated with the presentation of a TSCI.

Cardenas et al. in 2004 investigated re-hospitalization rates after traumatic spinal cord injury and found 55% are re-hospitalized in the first year and 37% are hospitalized per year afterwards [5]. Our results showed lower rates of re-hospitalization, but still a significant difference between the First Nations and non-First Nations cohorts. Among First Nations people with TSCI, 39% required hospitalization during the two-year follow-up period compared to 17% in the non-First Nations cohort. This again suggests that First Nations Peoples in Alberta living with TSCI are not getting sufficient care services in the community to prevent SCI-related complications and avoid re-hospitalizations, which also constitutes a health inequity against Indigenous Peoples in this province.

The NTSCI population generally had higher rates of utilization of all types of medical care compared to the TSCI group, which may be partially accounted for by greater number of comorbidities in these individuals as shown by the Charlson index. When comparing First Nations to non-First Nations cohorts with NTSCI, GP and specialist visits were found to be significantly more in the First Nations group with rate ratios of 1.4 and 1.6, respectively (Table 2). The utilization of ED, inpatient visits, and number of inpatient days between the NTSCI First Nations and non-First nations cohorts were not statistically significant; however, the estimated rate ratios still showed higher utilization of these services by the First Nations cohort (i.e., 1.6, 1.6, and 1.5, respectively), which may reflect limited power detect those differences due to the lower number of individuals included after case matching (Table 2). Regardless, these findings could also be interpreted as a demonstration that First Nations Peoples living with NTSCI in this province require higher health service utilization, again suggesting a gap in services to help them control their illness, demonstrating inequities in the quality of care they receive.

When analyzing aggregated cohort data, the First Nations cohort had higher rates of visits to GP, specialists, ED, inpatient visits, and inpatient days (Supplementary Fig. 1). Aggregated data for those living with SCI in rural locations found they had less GP and specialist visits when compared to those in urban or metropolitan centers. In contrast, ED visits were identified to be more frequent in rural SCI patients. This increased dependence on ED resources for healthcare in rural locations suggests another area for improvement by increasing support by GPs and specialists to facilitate care outside of major centers. Consequently, it is fair to interpret that the rural SCI population in Alberta is facing important inequities in accessing medical services that could decrease further complications and avoid the use of high-cost services, including ED visits or inpatient hospitalizations.

### Study limitations

First Nations identification was performed by the Alberta Ministry of Health based on the First Nations Status Registry, which will miss individuals without updated/valid registration. Using this method, we were not able to collect data for other Indigenous Peoples including Métis and Inuit populations, who are also at risk of facing the healthcare inequities identified in this study. Consequently, our findings can only be applied to the registered First Nations population of Alberta, and the observed prevalence estimates and rates of healthcare service utilization and complications could have been under or over estimated. Another limitation of this study is related to the utilization of administrative data, which did not allowed us to quantify the severity of SCI. Considering the severity of injury may help identify further differences between cohorts, offering further insights into their healthcare utilization.

## Conclusion

Significant differences exist in the prevalence of SCI between First Nations and non-First Nations Peoples living in Alberta, as well as differences in healthcare utilization that suggest the existence of healthcare inequities faced by First Nations Peoples living with SCI in this Canadian province. Further investigation into healthcare access and SCI support focused on Indigenous healthcare will be valuable for decreasing these inequities.

### Reporting summary

Further information on research design is available in the Nature Research Reporting Summary linked to this article.

## Supplementary information

Reporting Summary

Supplementary Material

Supplementary Material Legends
