# Supplementary material for: Epidemiology and healthcare utilization of First Nations peoples living with spinal cord injury in Alberta: an observational study to explore health inequities
Source: Spinal Cord Ser Cases. 2023 Sep 8;9:48. doi: 10.1038/s41394-023-00603-4 (PMC10484972; doi:10.1038/s41394-023-00603-4)
Supplement: Supplementary file 3 — Supplementary Material Legends [file 41394_2023_603_MOESM3_ESM.docx]

Supplementary Table 3: Number of Visits to Healthcare Before Case Matching

- Rate ratios and associated p values comparing visit rates between First Nations and non-First Nations populations were estimated through negative binomial modelling.

Supplementary Table 4: Rates of SCI Complications Before Case Matching

- Rate ratios and associated p values comparing complication rates between First Nations and non-First Nations populations were estimated through negative binomial modelling.

Supplementary Table 5: Complications by Sector and Type Before Case Matching

Rate of common SCI complications and type of healthcare access divided into

community (GP and Specialists), ED, and inpatient hospitalizations.

Supplementary Table 6: Complications by Sector and Type After Case Matching

Rate of common SCI complications and type of healthcare access divided into

community (GP and Specialists), ED, and inpatient hospitalizations.

Supplementary Figure 1: Rate Ratios for Healthcare Utilization

Rate ratios with 95% CI for aggregated cohort data based on demographics and location of healthcare access.
